# E-Cigarette Vapour Increases ACE2 and TMPRSS2 Expression in a Flavour- and Nicotine-Dependent Manner

**DOI:** 10.3390/ijerph192214955

**Published:** 2022-11-13

**Authors:** Rhys Hamon, Miranda P. Ween

**Affiliations:** 1 School of Medicine, Faculty of Health Sciences, University of Adelaide, Adelaide 5000, Australia; 2 Centre for Cancer Biology, University of South Australia and SA Pathology, Adelaide 5000, Australia; 3 Department of Thoracic Medicine, Royal Adelaide Hospital, Adelaide 5000, Australia

**Keywords:** COVID-19, coronavirus, E-cigarettes, nicotine, cigarette, ACE-2, TMPRSS2

## Abstract

COVID-19 infects via the respiratory system, but it can affect multiple systems and lead to multi system failure. There is growing evidence that smoking may be associated with higher rates of COVID-19 infections and worse outcomes due to increased levels of ACE2 in lung epithelial cells, but it is unknown whether E-cigarette use may lead to increased risk of COVID-19 infection from the SARS-CoV-2 virus. In this study, healthy donor bronchial epithelial cells (NHBE) and monocyte-derived macrophages (MDM) were exposed to cigarette smoke extract (CSE) or nicotine or flavoured E-cigarette vapour extract (EVE) before the assessment of SARS-CoV-2 recognition receptors ACE2 and TMPRSS2 genes. MDMs exposed to CSE and Tobacco EVE showed increased ACE2 expression; however, no treatment altered the TMPRSS2 expression. ACE2 was found to be upregulated by >2-fold in NHBE cells exposed to CSE, as well as nicotine, banana, or chocolate EVE, while TMPRSS2 was only upregulated by CSE or nicotine EVE exposure. These findings suggesting that flavourings can increase ACE2 expression in multiple cell types, while TMPRSS2 expression increases are limited to the epithelial cells in airways and may be limited to nicotine and/or cigarette smoke exposure. Therefore, increased risk of COVID-19 infection cannot be ruled out for vapers.

## 1. Introduction

COVID-19 has dominated the news for over two years, with infections in the hundreds of millions and rising, and killing millions worldwide. Researchers have been focussed on understanding how cells are infected with SARS-CoV-2 in an effort to understand how these infections could be blocked with treatments.

SARS-CoV-2 appears to initially infect the host through the respiratory tract, gaining access to other critical organs through the circulatory system, such as the liver, kidneys, heart, and gastrointestinal tract, leading to potential multi-organ failure [1,2,3,4]. The most well-known receptor that the virus binds to for entry into cells is angiotensin-converting enzyme 2 (ACE2) [5], an enzyme attached to the surface of cells. In normal physiology, it is a key player in the renin–angiotensin–aldosterone system (RAAS), which is a critical regulator of blood volume and systemic vascular resistance. ACE2 cleaves angiotensin I to angiotensin II which can then bind to angiotensin II type I (ATI) and type II (ATII) receptors in order to maintain extracellular volume and arterial blood pressure via plasma sodium concentration [6].

The SARS-CoV-2 spike (S) glycoprotein, which is involved in receptor recognition and viral attachment, is highly conserved among all human coronaviruses and is surface-exposed. Specifically, the S glycoprotein of SARS-CoV-2 harbours a Furin cleavage site that must be enzymatically cleaved to mediate entry into host cells, making it unique to SARS-CoVs and SARS-related coronaviruses. TMPRSS2 is known to cleave the S protein for cellular entry [7,8] and blocking TMPRSS2 activity inhibited the entry of SARS-CoV-2 into a range of cells from different origins, including lung cells, in vitro [9,10]. Briefly, the SARS-CoV-2 receptor-binding domain of the spike glycoprotein interacts with the tip of subdomain I of ACE2, facilitating membrane fusion between the virus and the host cell ACE2, allowing for viral RNA release into the host cell cytoplasm, infecting the cell. The binding affinity for SARS-CoV-2 has been shown to be much higher than SARS-CoV-2 [11], and spike mutations in some variants have been described as having an even greater binding affinity with ACE2 [12].

Within the lungs, ACE2 and TMPRSS2 are primarily expressed on secretory epithelial cells [8,13,14,15] and they are thus an area of focus in COVID research. However, the virus progresses beyond the lungs in many patients and can lead to multi-organ damage. Little is understood about how the virus spreads from the lungs to other tissues. Macrophages are able to migrate from the lungs to the blood stream and to other organs. There is some evidence that macrophages may play a role in helping to deliver other coronaviruses to tissues beyond the lungs [16,17], although this has not yet been proven to be the case with SARS-CoV-2. Macrophages and monocytes have been shown to express ACE2 [15,18,19], and some degree of differentiation of monocytes to macrophages has been observed in the circulation of those infected with SARS-CoV-2 [20]. Furthermore, an increased presence of monocyte-derived infiltrating macrophages in SARS-CoV-2-infected lungs has been observed during the acute inflammatory phase [21,22]. Thus, it has been proposed that macrophages may be one way that the virus could transit from the lungs to the rest of the body [23,24,25], and it may play a role in hyper-inflammation seen in patients with severe disease, including by producing T cell chemoattractant chemokines [22,26].

In addition to research understanding how SARS-CoV-2 enters human cells, there has also been a lot of research dedicated to identifying those who may be at increased risk of infection and severe outcomes from infection. There has been much speculation regarding whether smoking or vaping increases the rates of infection with SARS-CoV-2 leading to COVID infections, and whether these infections may be more serious. Identifying those most at risk of serious disease remains an important area of research, even with several vaccines now available. Several meta-analyses have reported that the data as a whole showed increased severity in smokers [27,28,29,30]. One possible cause that was identified for this was increased ACE2 in the epithelial cells in the airways of smokers vs. non-smokers [14,31,32,33]. However, a minority claimed that nicotine and/or smoking actually reduced COVID infection rates [34,35,36], which is contrary to studies into the effects of nicotine on ACE2 and its downstream pathways, as well as its role in increased lung disease development, that were conducted long before COVID-19 was even on the horizon [37,38]. One limitation of these studies investigating increased COVID rate and disease severity in smokers is that it is difficult to obtain an accurate history from patients who are either intubated or in respiratory failure.

Understandably, interest has turned to whether the use of E-cigarettes, or vaping, may also increase SARS-CoV-2 receptor expression and lead to increased infection rates and severity. However, research has been limited and has instead explored the impacts of nicotine, rather than the flavouring chemicals (flavourants) added to the E-liquids, which a growing body of evidence shows can have ill health effects without the presence of nicotine [39]. In this study, we exposed bronchial epithelial cells and monocyte-derived macrophages from healthy donors to extracts made from cigarette smoke, nicotine E-liquid only, propylene glycol and vegetable glycerine base alone (PG:VG), EVE from 4 flavoured, and nicotine free E-liquids to assess the effects of individual components within the E-liquid on SARS-CoV-2 receptors in both airway epithelial cells and macrophages.

## 2. Materials and Methods

### 2.1. EVE and CSE Preparation

E-cigarette vapour extract (EVE) was produced as previously described [40]. Using 50 × 3 s puffs, 1 puff per 10 s, in a 70% PG:30% VG base (PG:VG) and nicotine (at 18 mg/mL in PG:VG) were vaporised. The control medium (C) was obtained using the same system to pass air through the medium for the same duration as E-cigarette use. Cigarette smoke extract (CSE) was prepared, as previously described [40]. Mango, banana, and tobacco EVE were used at 100%, chocolate EVE was used at 50%, and CSE at 10%, in order to remove known toxicity effects [41].

### 2.2. Preparation of Primary Bronchial Epithelial Cells

Bronchial epithelial cells from healthy non-smokers were purchased prior to the start of the pandemic (NHBE normal human bronchial/tracheal CC-2540, Lonza, Norwest, NSW, Australia) and were cultured as per the manufacturer’s instructions (n = 8). The cells were seeded at 0.7 × 10^6^ in BEGM and given 24 h to reach confluence to encourage contact inhibition of proliferation, and the wells were then rinsed and treated for 24 h with EVE or CSE as previously described [42].

### 2.3. Monocyte-Derived Macrophages (MDM)

The healthy non-smoking, non-vaping donors all had no known respiratory illness (n = 6). Venous blood was collected in 10 U/mL preservative free sodium heparin tubes (Becton Dickinson, Mt Wellington, New Zelanad). The Royal Adelaide Hospital Human Research Ethics Committee approved the protocol and informed consent was obtained. Samples were collected before the start of the pandemic. MDM were differentiated culturing in RPMI 10% FCS and 2 ng/mL GM-CSF for 12 days, with the media changed on day 4 and day 8 to create “alveolar-like” macrophages [43], as previously published [44]. On day 13, MDM were exposed to EVE or CSE for 24 h.

### 2.4. Sample Collection and cDNA Conversion

The media were removed from the treated cells and the cells were collected in an RLT lysis buffer and the total RNA was extracted by RNeasy Plus mini kit (Qiagen, Chadstone Centre Victoria, Melbourne, VIC, Australia). Genomic DNA was removed via on column digestion with RNAse-free DNAse with the total RNA isolated as per the kit instructions. RNA was quantified on a Nanodrop spectrophotometer (Life Technologies, Mulgrave, VIC, Australia) and cDNA was prepared from 2 µg of total RNA using a high-capacity cDNA reverse transcription kit (Applied Biosystems, Mulgrave, VIC, Australia) with an RNase inhibitor (Applied Biosystems).

### 2.5. Taqman qPCR

Quantitative PCR was performed using 50 ng (NHBE) or 125 ng (MDM) of cDNA template in 10 µL reactions using TaqMan assays with the TaqMan gene expression master mix (Applied Biosystems) on a CFX96 Real-Time PCR system (Bio-rad). Angiotensin Converting Enzyme 2 (ACE2: Hs00222343_m1) and Transmembrane Serine Protease 2 (TMPRSS2: Hs00237175_m1) were normalised to housekeeping genes; Hypoxanthine guanine phosphoribosyl transferase (HPRT: Hs99999909_m1) and Glyceraldehyde-3-Phosphate Dehydrogenase (GAPDH: Hs99999905_m1) with the expression relative to the control samples calculated using the 2−ΔΔCt method and presented as Log2(Fold change).

### 2.6. Statistical Analysis

Statistical analysis was performed using GraphPad PRISM version 8. Data were checked for normality and outliers prior to analysis by repeated measure one-way ANOVA with Tukey’s multiple comparisons testing reporting adjusted *p*-values, with values <0.05 considered significant. Data are presented as mean and SEM with individual data points also graphed.

## 3. Results

### 3.1. MDM ACE2 and TMPRSS2 Expression

**Figure 1:**
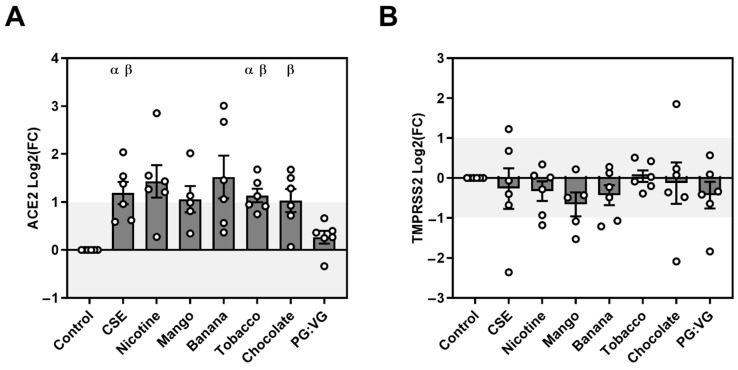
Relative expression of ACE2 and TMPRSS2 mRNA in exposed healthy monocyte-derived macrophages. Monocyte-derived macrophages from healthy donors were exposed to cigarette smoke extract or E-cigarette vapour extract for 24 h before mRNA extraction and cDNA conversion. Quantitative PCR was performed for (**A**) ACE2 and (**B**) TMPRSS2 and normalised to housekeeping genes HPRT1 and GAPDH. The expression relative to the control samples was calculated using the 2−ΔΔCt method and is presented as Log2(Fold change). N = 6 donors. α = *p* < 0.05 from control, β = *p* < 0.05 from PGVG (glycol base).

MDMs showed low levels of ACE2 and TMPRSS2 expression compared with the bronchial epithelial cells (data not shown). We observed flavour-dependent significant increases in ACE2 in healthy donors MDM exposed to EVE. In comparison with the air control, the CSE and tobacco EVE showed an increased ACE2 expression (2.43-fold and 2.25-fold, respectively), and compared with PG:VG:CSE, tobacco EVE and chocolate EVE showed a small but significant increase in expression (1.95-fold, 1.90-fold, and 1.74-fold, respectively) (Table 1, Figure 1). TMPRSS2 expression did not change with any treatment.

### 3.2. NHBE ACE2 and TMPRSS2 Expression

**Figure 2:**
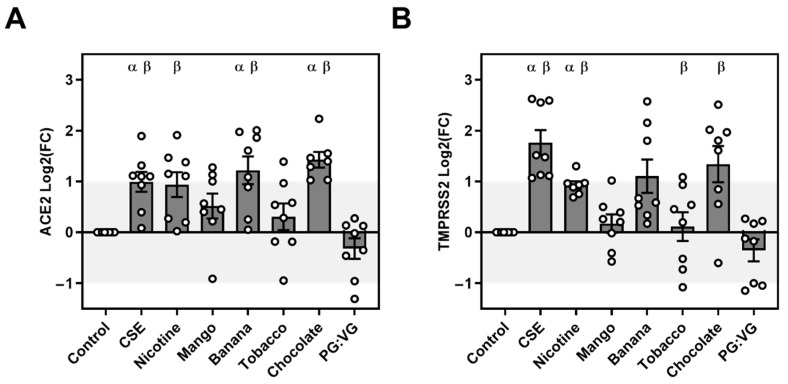
Relative expression of ACE2 and TMPRSS2 mRNA in exposed healthy NHBEs. Normal healthy bronchial epithelial (NHBE) cells from healthy donors were exposed to cigarette smoke extract or E-cigarette vapour extract for 24 h before mRNA extraction and cDNA conversion. Quantitative PCR was performed for (**A**) ACE2 and (**B**) TMPRSS2 and normalised to housekeeping genes HPRT1 and GAPDH. The expression relative to the control samples was calculated using the 2−ΔΔCt method and presented as Log2(Fold change). n = 8 donors. α = *p* < 0.05 from control, β = *p* < 0.05 from PGVG (glycol base).

We observed flavour-dependent significant increases in ACE2 and TMPRSS2 in the healthy donor NHBE exposed to EVE. In comparison with the air control, ACE2 increased 2.11-fold in CSE exposed to NHBE, 2.60-fold to banana, and 2.46-fold to chocolate EVE. In comparison with PG:VG, ACE2 increased 2.66-fold in CSE exposed to NHBE, 2.64-fold to nicotine, 3.23-fold to banana, and 3.07-fold to chocolate EVE. In comparison with air, TMPRSS2 expression in treated NHBE increased 3.77-fold with CSE, and 1.92-fold with nicotine EVE. When compared with PG:VG, the TMPRSS2 expression in the treated NHBE increased 5.78-fold with CSE, 2.76-fold with nicotine EVE, 1.4-fold with tobacco, and 4.07-fold with chocolate (Table 2, Figure 2).

## 4. Discussion

Underlying conditions have been front and centre in COVID discussions, with early studies seeking to identify those who may be at increased risk of infection or severe disease and death. While early data on diabetics and obese patients were rapidly accepted, early data showing smokers may be at heightened risk became controversial, in part, due to a few studies suggesting nicotine or smoking was actually protective against infection [34,35,36], despite the long and existing body of literature showing the effects of nicotine on the RAAS [37,38,45]. A handful of other studies also showed no difference in infection rates in smokers [46,47,48,49]. However, many studies have since been published supporting the idea that smokers were likely to be at heightened risk of disease severity [27,28,29,30,50,51,52]. This, in part, has been linked to the increased expression of the SARS-CoV-2 receptor, ACE2, observed in the lungs [13,15,31,32,33,53,54,55,56] or blood [57,58] of smokers. Studies investigating the expression of TMPRSS2, the enzyme responsible for cleaving the viral spike protein allowing it to bind to ACE2 in smokers, also revealed an increased expression [15,31,32,53,54,55,56]. Furthermore, it is known that smokers’ airways show increased inflammation [59], increased bacterial and viral respiratory infections [60], and that their macrophages show a reduced ability to clear viruses [61], as well as reported dysfunction [41,62,63]. Interestingly, monocyte-derived macrophages have been posited by several papers as a possible mechanism through which the virus may spread beyond the lungs [16,17] and may play a role in hyper-inflammation during infection, but while some studies have shown they may express ACE2 [15,64,65] and TMPRSS2 [66,67], there has been little investigation into whether smoking or vaping affected the expression of these genes in macrophages.

Given that E-cigarettes have also been shown to alter macrophage and neutrophil bacterial clearance [40,41,68], it is fair to question whether E-cigarettes may also induce changes in the expression of ACE2 and TMPRSS2 in the lungs, and potentially lead to increased rates of infection or increased infection severity. The studies that were published on infection rate and severity in vapers suffered from a flawed methodology. For example, Gaiha et al. used self-reported infection and showed that ever users had increased COVID diagnosis, but not past 30-day users, which appeared contradictory [69]. Jose et al. showed no difference in COVID diagnosis incidence in E-cigarette users [34], while Chen et al. showed increased self-reported COVID diagnosis in the dual users of tobacco cigarettes and E-cigarettes [54].

Thus, the answer to the question of whether E-cigarettes increased susceptibility to SARS-CoV-2 infection via increased ACE2 and TMPRSS2 and whether macrophages may also be affected remained “inconclusive”. In this study, we sought to establish the effects of cigarette smoke and E-cigarette vapour on the ACE2 and TMPRSS2 in healthy bronchial epithelial cells and monocyte-derived macrophages.

In this study, we observed that cigarette smoke exposure induced the expression of ACE2 in MDMs, but that nicotine alone was only trending towards an increase. Tobacco-flavoured EVE also increased the expression of ACE2, suggesting that the flavourings used in E-liquids could increase the risk of infection. We did not observe any increase in TMPRSS2 expression in the MDMs with any treatment, suggesting that smoking may only affect ACE2 expression and not the cleavage of the viral spike protein needed for binding to ACE2. Macrophages have been shown to be able to be infected by SARS-CoV-2 via multiple mechanisms, including general uptake and phagocytosis of infected damaged epithelial cells [23,25,26,70], and may play a role in transporting the virus around the body or play a role in inflammation in the lungs. Little work has been done in this area, but studies have shown that particulate matter (a known pathogenic component of cigarette smoke) increased ACE2 and TMPRSS2 expression in murine lung macrophages [66]. One study showed that ACE2 and TMPRSS2 was increased in CD45+ immune cells, which would include macrophages, of smokers and vapers [71]. Another study utilising a ferret SARS-CoV-2 infection model found that blood monocyte-derived macrophages were the most common infiltrated immune cells in the lungs, and the macrophages showed the presence of the virus. These macrophages also showed a high level of differential gene expression post infection versus no infection 34315893. In a human ACE2 transgenic mouse model, macrophages in the lungs were found to facilitate SARS-CoV-2 infection of the lungs [23].

In our NHBE cells, cigarette smoke exposure also increased the gene expression of ACE2, but in these cells, it also increased the expression of TMPRSS2. This adds to the body of growing evidence that smoking is linked to a higher chance of infection with SARS-CoV-2. One study exposed gingival epithelial cells to cigarette smoke and showed increased ACE2 and TMPRSS2, as well as increased internalisation of SARS-CoV-2 via AChR signalling, which was able to be blocked by AChR RNAi [72], showing a direct link between smoke exposure, increased ACE2 and TMPRSS2, and increased viral uptake.

As part of our study, we investigated the effect of vapour from flavourless nicotine freebase + PG:VG E-liquid on ACE2 and TMPRSS2 gene expression in NHBE cells. ACE2 was close to significant increases, and TMPRSS2 showed a significant increase in gene expression, while PG:VG only did not cause any change in the expression levels for either gene, showing that this effect to be from the nicotine alone. This suggests that nicotine may be one of the components in cigarette smoke causing this increased expression. However, this also raises concerns for vapers using nicotine. Nicotine is well known to up-regulate the ACE/angiotensin-II/ATI axis and down-regulate the compensatory ACE2/ANG-(1–7)/Mas receptor axis, a change which is known to contribute to cardiovascular and pulmonary diseases [38,73,74]. Bronchial epithelial cells express nicotinic acetylcholine receptors (nAChR), specifically the α-3,5, and 7 subtypes [75,76].

One study showed that female mice exposed to PG+ Nicotine 5 days/week for a month had increased lung ACE2, but when the nicotine receptor α7 nAChR was knocked out, there was no increase in ACE2. Interestingly, this seemed to affect female mice, but not male mice [77]. Another study found that in male lungs, nicotine vapor inhalation induced a significant increase in ACE2 mRNA and protein, but in this case, these differences were not found in females [78]. Both vehicle and nicotine vapor inhalation downregulated α5 nAChR subunits in both sexes, while differences were not found in the α7 nAChR subunit expression [78]. A study by Naidu et al. exposed mice to base + nicotine daily for 3 weeks and found that the whole lung ACE2 expression increased both with and without nicotine, but the increases were greater in the male mice compared with the female mice and the addition of nicotine significantly increased over glycol base alone [79]. The fact that more men smoke than women in most countries has been posited as a reason that men overall have a worse outcome than women [80,81,82].

Thus, there may be gender-specific nicotine-dependent results that should be considered when considering vapers, which have allowed whole of population studies to find no increases in E-cigarette users as they have not broken down their results by gender [34,46,83]. Furthermore, if additional effects are flavour-dependent, increases in users using those flavours can easily be masked by those who are not. In comparison, cigarette smoke may affect infection rates via additional mechanisms to nicotine.

One study using a lung epithelial cell line had inconsistent findings on the effects of E-cigarettes on ACE2 expression, with PGVG increasing ACE2, but not PGVG+ nicotine, and the testing of a single flavour containing PG:VG not showing an increase, but the flavour + nicotine had an increase in ACE2 by PCR, making it hard to draw any conclusions from this work [84]. Another study investigated ACE2 and TMPRSS2 expression in bronchial brushings from vaper, but this may have been confounded by all of the users being ex-smokers [54]. We thus chose to expose human bronchial epithelial cells from healthy never smoking donors to four different flavoured E-liquids (confirmed nicotine free [41]) and PGVG base alone, and to assess ACE2 and TMPRSS2 by qPCR. We observed that banana and chocolate both increased the ACE2 expression, but not tobacco or mango flavours, and that none of the flavours tested increased the TMPRSS2 expression. Our previous studies have shown that the banana- and chocolate-flavoured E-liquids contained high levels of Benzene-ring containing flavouring chemicals, while the tobacco and mango did not [41]. This is a possible mechanism for the increased ACE2, which could be investigated further in future studies. The flavour specific finds may also help to explain the contradictory findings in humans, as there are thousands of flavours available for sale, and depending on the distribution of the flavour usage in the cohort, flavourings that may affect ACE-2 and TMPRSS2 may be masked by flavours that do not.

This work helps to support the findings of a handful of other studies that have suggested a link between E-cigarette use, ACE2 expression, and COVID. The first of which was a study where unflavoured nicotine E-cigarette vapour exposed mice had a worse infection with a mouse coronavirus and increased inflammation [85]. Masso-Silva et al. exposed mice to two flavours plus nicotine salts (Juul) daily for at least a month. They found increased ACE2 in the lungs of mint-exposed mice but not mango, suggesting a possible flavour effect [85]. Another study had often self-contradictory findings, but nevertheless suggested increased ACE2 in a lung epithelial cell line exposed to a single flavour of E-cigarette vapour condensate [84]. Our work, combined with these studies, suggest a pathway for more severe COVID infections in vapers, as has been documented in smokers, linked to an increased ACE2 expression.

### Limitations

This study was performed using an *in vitro* model, and may not reflect the true situation *in vivo.* No protein studies were performed to validate the mRNA findings; however, previous studies have shown that mRNA levels did correspond with protein in the airways [33,78]. Additionally, only four flavours were tested, and there are thousands of E-liquid flavours for sale globally, made up of various combinations of chemical flavourings and varying concentrations, so it cannot be definitively said that all flavours will not induce an ACE2 upregulation in airway cells. However, our findings show that some of the flavoured, nicotine-free EVE could induce an upregulation of TMPRSS2 in the donor NHBEs, suggesting that certain chemical flavourings may put users at increased risk of COVID infection. Our donors were predominantly male (HBE—2F, 6M, MDM—2F, 4M), which may show that our effects may be limited to or enhanced in men.

## 5. Conclusions

Given the evidence in this study and others, it is fair to hypothesise that both smoking and vaping may increase the expression of SARS-CoV-2 receptors on the surface of cells within the lungs, the initial point of entry for the virus, which may in turn lead to increased susceptibility to infection and/or worse outcomes. This study provides additional evidence to support the role of nicotine in SARS-CoV-2 receptor expression, and suggests that one reason for conflicting reports in the literature on vaping and COVID rates/severity may be due to the effects of specific flavours on receptor expression.

## Figures and Tables

**Table 1:** Comparison of control and PGVG (glycol base) E-cigarette vapour extract exposed monocyte-derived macrophage (MDM) ACE2 and TMPRSS2 mRNA with cigarette smoke extract and flavoured E-cigarette vapour extract.

		MDM ACE2 mRNA Expression				MDM TMPRSS2 mRNA Expression			
Tukey’s Multiple		Fold Change	Log2(FC)	Adjusted *p* Value		Fold Change	Log2(FC)	Adjusted *p* Value	
Comparisons Test		Mean(SEM)	Mean(SEM)			Mean(SEM)	Mean(SEM)		
PG:VG vs.	Control	1.23(0.10)	0.26(1.06)	0.570	ns	0.83(0.17)	−0.43(0.73)	0.869	ns
CSE vs.	Control	2.43(0.41)	1.18(1.09)	**0.035**	*	1.08(0.31)	−0.27(1.06)	0.999	ns
	PG:VG	1.95(0.21)		**0.027**	*	1.42(0.43)		1.000	ns
Nicotine EVE vs.	Control	3.12(0.85)	1.43(0.90)	0.073	ns	0.93(0.11)	−0.33(1.02)	0.850	ns
	PG:VG	2.55(0.70)		0.093	ns	1.23(0.22)		0.999	ns
Mango EVE vs	Control	2.24(0.47)	1.34(1.27)	0.113	ns	0.69(0.14)	0.08(0.63)	0.439	ns
	PG:VG	1.67(0.47)		0.343	ns	1.05(0.17)		0.986	ns
Banana EVE vs.	Control	3.66(1.16)	1.52(0.89)	0.154	ns	0.80(0.13)	−0.43(1.03)	0.675	ns
	PG:VG	3.42(1.27)		0.431	ns	1.31(0.40)		0.999	ns
Tobacco EVE vs.	Control	2.25(0.23)	1.13(1.17)	**0.005**	**	1.06(0.11)	0.05(0.81)	0.999	ns
	PG:VG	1.90(0.23)		**0.046**	*	1.73(0.62)		0.896	ns
Chocolate EVE vs.	Control	2.17(0.32)	1.03(1.02)	0.070	ns	1.26(0.49)	−0.13(1.21)	0.999	ns
	PG:VG	1.74(0.18)		**0.027**	*	1.71(0.46)		0.999	ns

* Represents *p* < 0.05; ** *p* < 0.01; Bold indicates < 0.05.

**Table 2:** Comparison of control and PGVG (glycol base) E-cigarette vapour extract exposed normal human bronchial epithelial (NHBE) cell ACE2 and TMPRSS2 mRNA with cigarette smoke extract and flavoured E-cigarette smoke extract.

		NHBE ACE2 mRNA Expression				NHBE TMPRSS2 mRNA Expression			
Tukey’s Multiple		Fold Change	Log2 (FC)	Adjusted *p* Value		Fold Change	Log2 (FC)	Adjusted *p* Value	
Comparisons Test		Mean(SEM)	Mean(SEM)			Mean(SEM)	Mean(SEM)		
PG:VG vs.	Control	0.85(0.10)	−0.32(0.42)	0.741	Ns	0.84(0.12)	−0.35(0.99)	0.718	ns
CSE vs.	Control	2.11(0.28)	0.99(0.34)	**0.018**	*	3.77(0.71)	1.76(0.85)	**0.003**	**
	PG:VG	2.66(0.41)		**0.004**	**	5.78(1.93)		**0.016**	*
Nicotine EVE vs.	Control	2.11(0.34)	0.94(0.40)	0.068	Ns	1.92(0.11)	1.16(1.07)	**0.0002**	***
	PG:VG	2.64(0.44)		**0.019**	*	2.76(0.43)		**0.013**	*
Mango EVE vs.	Control	1.57(0.25)	0.52(0.35)	0.484	Ns	1.19(0.16)	0.17(1.00)	0.969	ns
	PG:VG	2.05(0.38)		0.098	Ns	1.65(0.48)		0.497	ns
Banana EVE vs.	Control	2.60(0.43)	1.22(0.47)	**0.034**	*	2.60(0.60)	1.10(0.82)	0.119	ns
	PG:VG	3.23(0.50)		**0.011**	*	3.51(1.10)		0.073	ns
Tobacco EVE vs.	Control	1.38(0.24)	0.30(0.50)	0.918	Ns	1.22(0.23)	0.11(0.98)	1.000	Ns
	PG:VG	1.62(0.19)		0.100	Ns	1.4(0.08)		**0.034**	*
Chocolate EVE vs.	Control	2.46(0.42)	1.39(0.44)	**0.0009**	***	3.02(0.62)	1.34(0.77)	0.076	Ns
	PG:VG	3.07(0.66)		**0.006**	**	4.07(0.93)		**0.035**	*

* Represents *p* < 0.05; ** *p* < 0.01; *** *p* < 0.001; Bold indicates < 0.05.

## Data Availability

Data are contained within the article.
